# The absence of retinal input disrupts the development of cholinergic brainstem projections in the mouse dorsal lateral geniculate nucleus

**DOI:** 10.1186/s13064-018-0124-7

**Published:** 2018-12-12

**Authors:** Guela Sokhadze, Tania A. Seabrook, William Guido

**Affiliations:** Department of Anatomical Sciences and Neurobiology, University of Louisville School of Medicine, 511 S. Floyd St, Louisville, KY 40292 USA

**Keywords:** Retinogeniculate, Dorsal lateral geniculate nucleus, Acetylcholine, Parabigeminal nucleus, Cholinergic tegmentum, *math5-*null

## Abstract

**Background:**

The dorsal lateral geniculate nucleus (dLGN) of the mouse has become a model system for understanding thalamic circuit assembly. While the development of retinal projections to dLGN has been a topic of extensive inquiry, how and when nonretinal projections innervate this nucleus remains largely unexplored. In this study, we examined the development of a major nonretinal projection to dLGN, the ascending input arising from cholinergic neurons of the brainstem. To visualize these projections, we used a transgenic mouse line that expresses red fluorescent protein exclusively in cholinergic neurons. To assess whether retinal input regulates the timing and pattern of cholinergic innervation of dLGN, we utilized the *math5*-null (*math5*^−/−^) mouse, which lacks retinofugal projections due to a failure of retinal ganglion cell differentiation.

**Results:**

Cholinergic brainstem innervation of dLGN began at the end of the first postnatal week, increased steadily with age, and reached an adult-like pattern by the end of the first postnatal month. The absence of retinal input led to a disruption in the trajectory, rate, and pattern of cholinergic innervation of dLGN. Anatomical tracing experiments reveal these disruptions were linked to cholinergic projections from parabigeminal nucleus, which normally traverse and reach dLGN through the optic tract.

**Conclusions:**

The late postnatal arrival of cholinergic projections to dLGN and their regulation by retinal signaling provides additional support for the existence of a conserved developmental plan whereby retinal input regulates the timing and sequencing of nonretinal projections to dLGN.

## Background

The dorsal lateral geniculate nucleus (dLGN) of the mouse has become a model system to study the development of thalamic circuits [1–4]. Much of our present understanding is based on studies focused on the retinogeniculate pathway, the connections between retinal ganglion cells (RGCs) and dLGN neurons. While retinal projections provide the primary excitatory drive for relay neurons of dLGN, the vast majority of input is nonretinal in origin [5], and acts to modulate the gain of retinogeniculate transmission in a state-dependent manner [6, 7]. The primary sources of nonretinal input to dLGN include glutamatergic neurons of visual cortex layer VI, GABAergic neurons of the thalamic reticular nucleus, and the cholinergic neurons from different brainstem nuclei. Despite the fact that over 90% of all synapses in dLGN arise from nonretinal sources, we know little about how and when these projections innervate dLGN, or how they interact with the arrival and refinement of retinal projections. What little we do know is based on the corticothalamic pathway [8–10]. Layer VI neurons of the neocortex begin to innervate the dorsal thalamus at perinatal ages, but corticogeniculate innervation occurs largely after the first postnatal week, after the arrival of retinal axons and their refinement into non-overlapping eye-specific domains [9]. Moreover, retinal input orchestrates the timing of corticogeniculate innervation by regulating the levels of aggrecan, a repulsive extracellular matrix molecule [11]. What remains to be tested is whether such sequencing and reliance on retinal input is part of a conserved developmental plan that governs the arrival of other nonretinal inputs to dLGN.

To address this, we examined another major nonretinal projection to dLGN, the ascending cholinergic input from the brainstem. Estimates reveal that about 25% of all synapses in dLGN arise from brainstem cholinergic nuclei [12]. These projections have a substantial influence on retinogeniculate transmission, regulating the firing mode of dLGN neurons [13], establishing network states during sleep, wakefulness, and arousal [14, 15], as well as modulating visuo-motor interactions [16]. Previous immunohistochemical studies have shown a late postnatal onset for the labeling of acetylcholine synthesizing enzyme (choline acetyltransferase, ChAT) in dLGN, which increases in density over a protracted period of development [17, 18]. However, little is known about the source, trajectory, and pattern of cholinergic innervation in the developing mouse dLGN. In several mammalian species, ascending cholinergic projections to dLGN originate from two distinct brainstem groups, the pedunculopontine and laterodorsal tegmental nuclei (PPTg, & LDTg), as well as the parabigeminal nucleus (PBG) [19–21]. The projection from PBG is especially notable since these axons course within the optic tract en route to dLGN and superior colliculus [20, 22, 23], raising the possibility that retinal axons participate in the guidance of PBG axons.

Here, we examined the postnatal development of cholinergic input to dLGN and tested whether the absence of retinogeniculate projections affects the timing and patterning of cholinergic innervation. To visualize ascending cholinergic projections, we crossed a ChAT-Cre knock-in mouse line with a Cre-dependent reporter strain (Ai9) to selectively drive expression of the fluorescent protein tdTomato (tdT) in cholinergic neurons [24–29]. To evaluate the development of cholinergic innervation of dLGN in the absence of retinal projections, we utilized a mutant mouse that lacks *math5*, a transcription factor necessary for RGC progenitor cell differentiation [30]. *Math5*^−/−^ mice exhibit a > 95% loss of RGCs, and the surviving RGCs fail to form an optic nerve, thus leaving the brain devoid of retinal input [31–34]. Furthermore, we used Cre-dependent viral tracing techniques to assess whether the trajectory of optic tract-associated PBG axons to dLGN was disrupted in a brain lacking retinofugal projections.

## Materials and methods

### Subjects

All breeding and experimental procedures were approved by the University of Louisville Institutional Animal Care and Use Committee. Transgenic mouse strains ChAT-IRES-Cre (Jackson Labs, stock #006410, strain B6;129S6-Chat^tm2(cre)Lowl^/J), Ai9 (Jackson Labs, stock #007909, strain B6.CgGt(ROSA)26Sor^tm9(CAG-tdTomato)HZe^/J), and *math5*^−/−^ (on a mixed C57B6/J and 129/SvEV background [9, 30, 32]) of either sex were used for breeding or for experiments. Homozygous ChAT-Cre mice were bred with homozygous Ai9 mice to generate ChAT-Cre x Ai9 offspring. ChAT-Cre^+/+^ x *math5*^−/−^ mice were bred with Ai9^+/+^ x *math5*^−/−^ mice to generate ChAT-Cre^+/−^ x Ai9^+/−^ x *math5*^−/−^ offspring.

To genotype for Cre, the following primers were used in polymerase chain reaction reactions (PCR): Cre-F (CCTTCTATCGCCTTCTTGACG), Cre-R (AGATAGATAATGAGAGGCTC), WT-F (GTTTGCAGAAGCGGTGGG), WT-R (AGATAGATAATGAGAGGCTC). PCR amplification was performed in 28 cycles by denaturation at 94 °C for 15 s, annealing at 60 °C for 15 s, and elongation at 72 °C for 10 s. To genotype for *math5*, the following primers were used: Neo-F (GCCGGCCACAGTCGATGAATC), Neo-R (CATTGAACAAGATGGATTGCA), *math5*-F (ATGGCGCTCAGCTACATCAT), and *math5*-R (GGGTCTACCTGGAGCCTAGC). PCR amplification was performed in 35 cycles by denaturation at 94 °C for 30 s, annealing at 59 °C for 30 s, and elongation at 72 °C for 45 s.

### Histology

To collect brain tissue for analysis, mice were deeply anaesthetized by hypothermia (<P5) or isoflurane vapors, and transcardially perfused with phosphate-buffered saline (PBS, 0.01 M phosphate buffer with 0.9% NaCl) followed by 4% paraformaldehyde (PFA) in 0.1 M phosphate buffer. Brains were postfixed overnight in 4% PFA, then transferred to PBS. A vibratome (Leica VT1000S) was used to make 70 μm-thick sections in the coronal plane. To amplify tdT fluorescence signal and prevent photobleaching during confocal imaging, DsRed (Clontech) antibody was applied using the following procedure: Sections were placed in blocking medium (10% normal goat serum (NGS), and 0.3% Triton X-100 in PBS) for 1 h, and then incubated for 12 h in rabbit anti-DsRed (1:1000) with 1% NGS in PBS. Next, tissue was incubated for 1 h in 1:100 biotinylated goat anti-rabbit IgG antibody (Vector Labs) with 1% NGS in PBS, followed by 1 h in 1:100 streptavidin Alexa Fluor (AF) 546 (Life Technologies) in PBS. All sections were mounted onto gelatin subbed glass slides using ProLong mounting medium containing DAPI (Life Technologies).

### CTB injection

Intravitreal binocular eye injections of the anterograde tracer cholera toxin subunit B (CTB) conjugated to Alexa Fluor 488 (Invitrogen) were done in an adult (P60) ChAT-Cre x Ai9 mouse. Under anesthesia (a mixture of ketamine, 120–140 mg/kg, and xylazine, 12–14 mg/kg), the sclera was pierced with a sharp-tipped glass pipette in order to drain excess vitreous. Another pipette, filled with 1% solution of CTB-AF-488 dissolved in distilled water, was inserted into the opening created by the first pipette. A picospritzer attached to the pipette was used to deliver approximately 2–3 μl of the CTB solution into the vitreous of the eye. After a 48-h survival period, brain tissue was harvested using the methods describe above.

### Viral tracer injection

Intracranial injections of a Cre-dependent adeno-associated viral tracer Flex-rev-oChIEF-tdTomato (Addgene plasmid #30541, serotype 9) were made in the left PBG of adult (>P60) ChAT-Cre and ChAT-Cre x *math5*^−/−^ mice. Prior to surgery, mice were deeply anesthetized using a mixture of ketamine/xylazine and head-fixed in a stereotaxic apparatus. An incision was made along the scalp, and a hole was drilled in the skull above the injection site (− 4.2 mm AP, − 1.75 mm ML from Bregma). A Hamilton syringe (World Precision Instruments) was guided by a stereotaxic apparatus, and used to deliver 15 nL of virus into the left PBG. After a 14–17 day incubation period, brain tissue was collected for histology using aforementioned procedures.

### Imaging and analysis

**Fig. 1:**
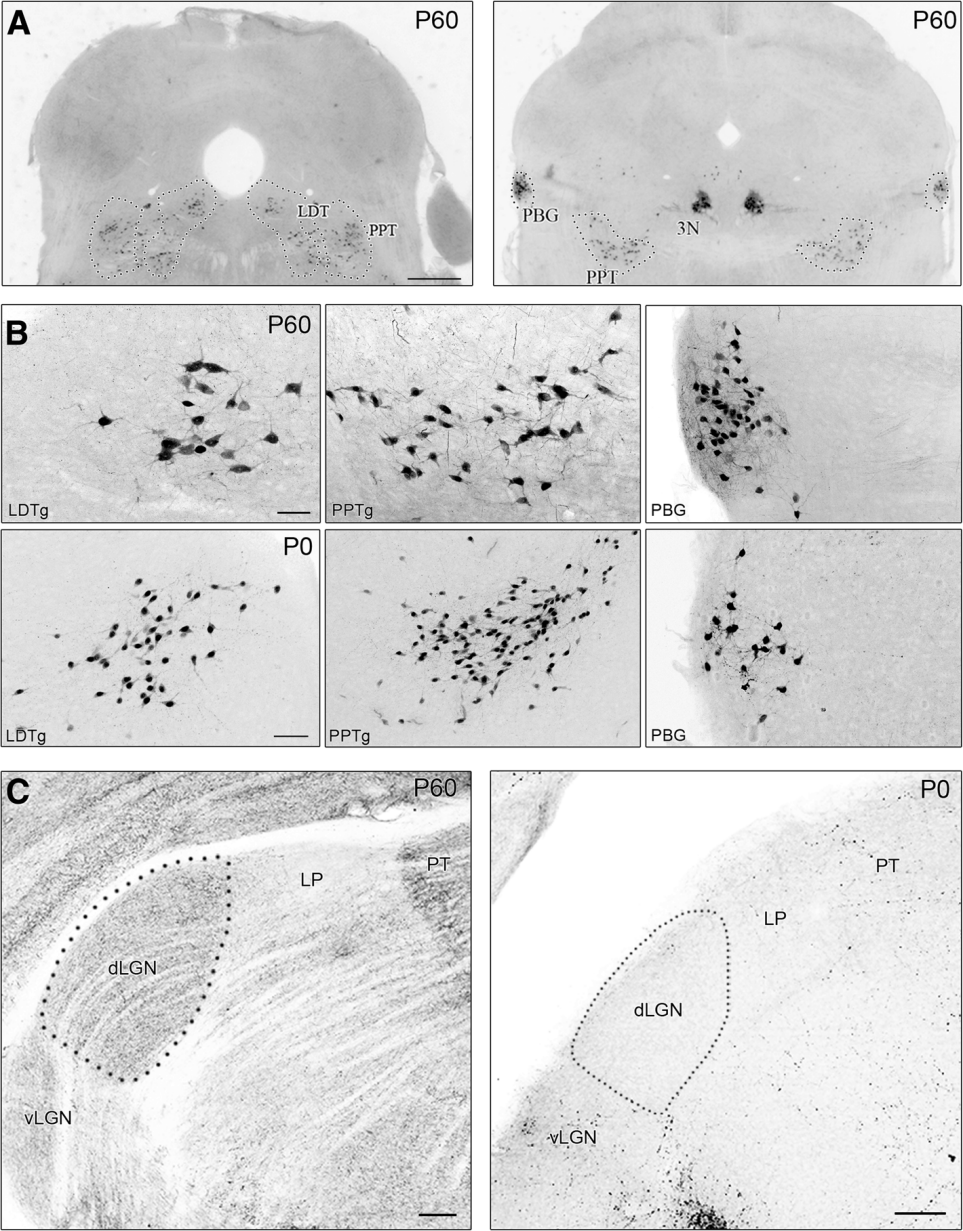
Pattern of tdTomato (tdT) labeling in brainstem and thalamus of ChAT-Cre x Ai9 mouse. **a** Coronal sections of the brainstem showing tdT labeling in cholinergic neurons in a P60 adult mouse. Left panel depicts the laterodorsal tegmentum (LDTg), and caudal region of pedunculopontine tegmentum (PPTg), and the right panel shows the rostral PPTg, parabigeminal nucleus (PBG), and oculomotor nucleus (3 N). Scale bars = 500 μm. **b** High power views showing tdT fluorescence in cholinergic neurons of LDTg, PPTg, and PBG of an adult (P60, top) and neonate (P0, bottom). Scale bars = 50 μm. **c** Coronal sections of adult (left) and neonate (right) dorsal thalamus showing tdT-labeled cholinergic projections and terminals arbors in ventral and dorsal lateral geniculate nuclei (vLGN, dLGN), lateral posterior nucleus (LP), and pretectum (PT). Dotted lines delineate the borders of the dLGN. In the adult, cholinergic fibers project extensively throughout the dorsal thalamus however, at P0 the dLGN is devoid of cholinergic input. Scale bars = 100 μm

All images were acquired on a confocal microscope (Olympus FV12000BX61) using Fluoview software. To acquire images of dLGN, a 20x objective lens (0.75 NA) was used to scan successive optical sections (1.14 μm step size), which were then collapsed to generate a Z-stacked image. In cases where the dLGN was larger than the area that could be captured using the 20x objective, two images were digitally stitched together to generate a single composite image (e.g. Fig. 4 WT P21 & P30). Acquisition parameters were calibrated using samples with highest and lowest levels of fluorescence to avoid oversaturation while maximizing detection. Black and white confocal images were inverted for clarity (e.g., Figs. 1, 3, 4, 6, 7, 8, 10, and 11). The heat maps depicted in Fig. 7 were generated using ImageJ (NIH) “thermal” lookup table function. When acquiring images to be used for quantitative analyses, acquisition parameters were kept consistent across samples. To quantify the degree of cholinergic innervation in dLGN, z-stack confocal images were imported into Photoshop (Adobe) and the area of dLGN was outlined with the aid of DAPI staining. A threshold value was chosen on the histogram that provided a clear distinction between signal and background (see also [9, 35–37]). Binarized images were generated and imported into ImageJ (NIH), where and the number of pixels comprising the fluorescence signal, as well as the number of total pixels representing the total area of dLGN were counted. For each section the degree of cholinergic innervation was expressed as the percentage of the fluorescence signal in relation to the total area of dLGN. For each hemisphere, 3–5 successive sections through the middle of dLGN were averaged to obtain the final value. For each age, 4–6 hemispheres were analyzed from 3 animals. To quantify the degree of innervation in the thalamic reticular nucleus (TRN), ventral posterior nucleus (VPL), and posterior nucleus (Po), similar procedures and measurements were made, but from a 200 × 200 μm region of interest (Fig. 6a).

To quantify the distribution of virally-labeled PBG arbors, sections containing dLGN were imaged using confocal microscopy, and then binarized using the threshold procedure described above. In each section, two lines were drawn through the middle of dLGN, dividing the nucleus into 4 equal quadrants (dorsomedial, DM; ventromedial, VM; dorsolateral, DL; ventrolateral, VL; Fig.12 inset). ImageJ was used to count the number of fluorescent pixels found in each of the quadrants. Values in each quadrant reflect a percentage of the total fluorescence detected throughout the entire nucleus. Each point depicts the average value taken from 4 to 5 sections of a single hemisphere.

To estimate the number of tdT-labeled PBG neurons in adult WT and *math5*^−/−^ ChAT-Cre x Ai9 mice, three 20-μm thick sections through the middle of PBG were imaged using confocal microscopy and each tdT-labeled soma was marked and quantified using ImageJ “Cell counter” function. To quantify the total number of PBG neurons labeled by FLEX-AAV-ChIEF-tdT virus, 70 μm-thick coronal sections through the rostro-caudal extent of PBG were used.

## Results

To visualize brainstem cholinergic neurons and their projections to dLGN, we crossed ChAT-Cre mice with an Ai9 reporter line [24–29]. Figure 1a shows the pattern of tdTomato (tdT) labeling in the brainstem in an adult (P60) ChAT-Cre x Ai9 mouse. Somatic labeling of tdT was seen in cholinergic neurons of brainstem nuclei reported to project to dLGN [17, 20, 22, 38], including laterodorsal tegmentum (LDTg, Fig. 1a, left), pedunculopontine tegmentum (PPTg, Fig. 1a, left & right), and parabigeminal nucleus (PBG, Fig. 1a, right). High power views of these nuclei (Fig. 1b) showed that tdT-labeled somata were present in the adult (P60) and at birth (P0), and comparable numbers of neurons were labeled. In adults, labeled projections were evident throughout the dorsal thalamus, including the ventral (vLGN) and dorsal lateral geniculate nuclei (dLGN), lateral posterior nucleus (LP), and in the pretectum (PT) (Fig. 1c, left). At P0, cholinergic innervation was sparse in these regions, and entirely lacking in dLGN (Fig. 1c, right).

**Fig. 2:**
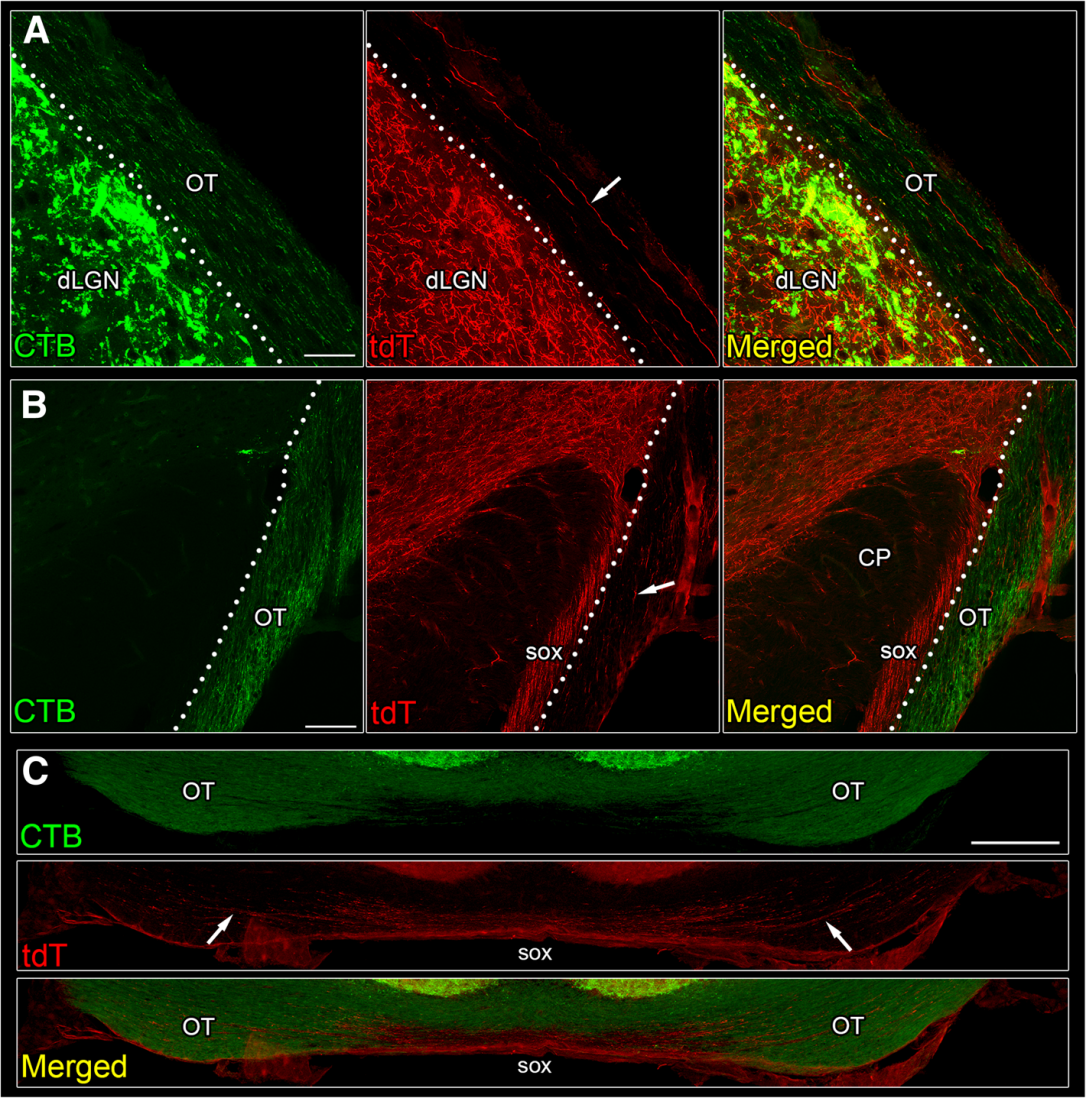
Relationship between cholinergic projections and the optic tract (OT). Coronal sections depicting OT (labeled with CTB-AF-488, green), cholinergic axons (labeled with tdT, red), and a merged image at the level of dLGN (A), cerebral peduncle (B), and optic chiasm (C). **a** Cholinergic axons course within OT along the dorsolateral border of dLGN. Dotted line delineates the border between the OT and dLGN. Scale bar = 30 μm. **b** Cholinergic axons travel within the supraoptic decussation (SOX) along the lateral edge of the cerebral peduncle (CP). Lateral to SOX, cholinergic axons also are seen coursing through OT. Dotted line delineates the border between SOX and OT. Scale bar = 30 μm. **c** Cholinergic axons are found along midline in SOX, just below the optic chiasm (OX) and also laterally in OT. Scale bar = 200 μm. In panels A-C, arrows depict location of cholinergic axons within OT

Consistent with observations made in other mammalian species [20, 23] in mouse we noted that (tdT-labeled) cholinergic axons traversed through the supraoptic decussation and the optic tract (Fig. 2). The supraoptic decussation contains multiple axonal tracts of nonretinal origin, lies adjacent to the optic tract near the cerebral peduncle, and crosses midline along the ventral border of the hypothalamus just caudal and ventral to the optic chiasm [39]. To distinguish between tdT-labeled cholinergic brainstem axons and retinal axons in the optic tract, we made binocular intravitreal injections of anterograde tracer cholera toxin subunit B (CTB) conjugated to Alexa Fluor 488 (green) in an adult ChAT-Cre x Ai9 mouse. Figure 2 provides coronal views of the optic tract at the level of dLGN (Fig. 2a), cerebral peduncle (Fig. 2b), and optic chiasm (Fig. 2c). At all levels, tdT-labeled cholinergic (red) and CTB-labeled retinal (green) axons shared similar trajectories. In the dorsal thalamus (Fig. 2a), cholinergic and retinal axons coursed together in the optic tract along the dorsolateral boundary of dLGN. At more ventral levels, cholinergic axons were found within the supraoptic decussation along the lateral edge of the cerebral peduncle, as well as in the adjacent optic tract (Fig. 2b). At the base of the brain, at the level of the optic chiasm, cholinergic axons were present along midline in the supraoptic decussation and more laterally where they comingled with retinal axons in the optic tract (Fig. 2c, Fig. 10).

**Fig. 3:**
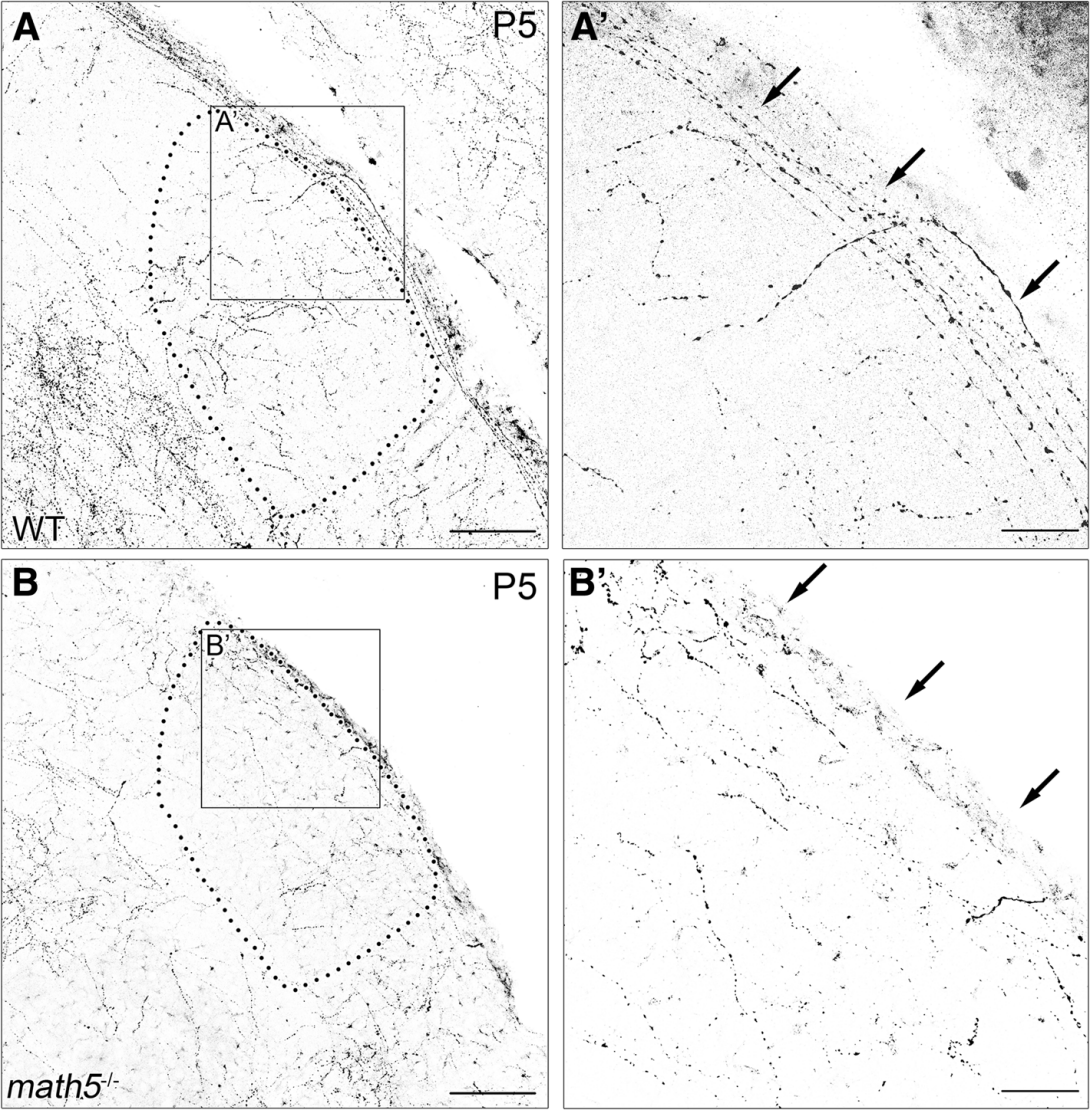
Arrival of cholinergic axons in dLGN during development in WT and *math5*^−/−^ mice. **a** Coronal section of WT dLGN at P5, showing cholinergic axons coursing along the dorsolateral edge and entering the nucleus (A’, arrows). **b** Coronal section of P5 dLGN in *math5*^−/−^. Cholinergic fibers are present in *math5*^−/−^ but are more widespread running through anteromedial aspect of dLGN (B’). Arrows highlight the differences in fiber trajectory. Dotted lines outline the boundaries of dLGN. Scale bars = 100 μm (left panels), 30 μm (right panels)

To determine whether retinal input is involved in regulating the development of cholinergic projections to dLGN, we compared the pattern and time course of innervation in ChAT-Cre x Ai9 mice (wild type, WT) with age-matched ChAT-Cre x Ai9 mice bred on a *math5*-null background (*math5*^−/−^), a mutant strain that lacks an optic nerve as well as central retinal projections [31, 32]. In both WT and *math5*^−/−^ mice, cholinergic fibers began to innervate dLGN by P5 (Fig. 3a and b). In WT, cholinergic axons closely followed the optic tract, innervating dorsolateral edge of dLGN (Fig. 3a and A’). However, in dLGN of *math5*^−/−^ mice (4 hemispheres at P5) the aggregation of cholinergic axons along the dorsolateral edge was less striking (Fig. 3B and B’).

**Fig. 4:**
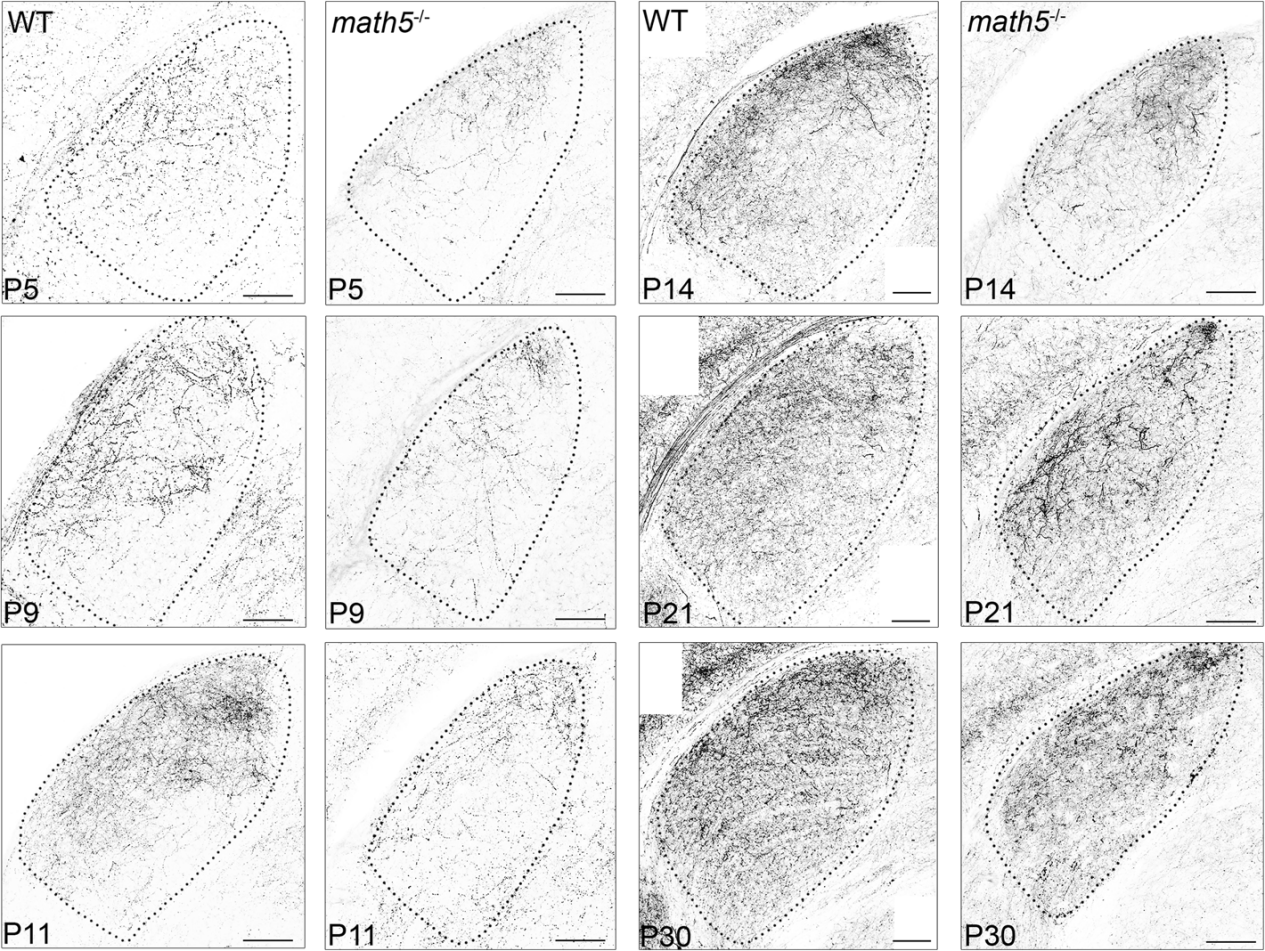
Cholinergic innervation of dLGN in the presence and absence (*math5*^−/−^) of retinal input at different postnatal ages. Examples of coronal sections at P5, P9, P11, P14, P21, and P30, showing the extent of cholinergic innervation in dLGN of WT (left) and *math5*^−/−^ mice (right). Dotted lines outline the boundaries of dLGN. Scale bars = 100 μm

Figure 4 shows the progression of cholinergic innervation in WT and *math5*^−/−^ at different postnatal ages (P5, P9, P11, P14, P21, and P30). As reported previously, there was a reduction in total size of dLGN in *math5*^−/−^ mice, compared to WTs [32]. In WTs between P5-P30, cholinergic innervation progressed steadily in a dorsolateral to ventromedial manner so that by P30 the entire nucleus contained a diffuse arrangement of cholinergic arbors. *Math5*^−/−^ mice also showed an age-related increase in cholinergic innervation. However, compared to WT, *math5*^−/−^ mice exhibited alterations in the rate and route of innervation, as well as in the patterning of their axon arbors.

**Fig. 5:**
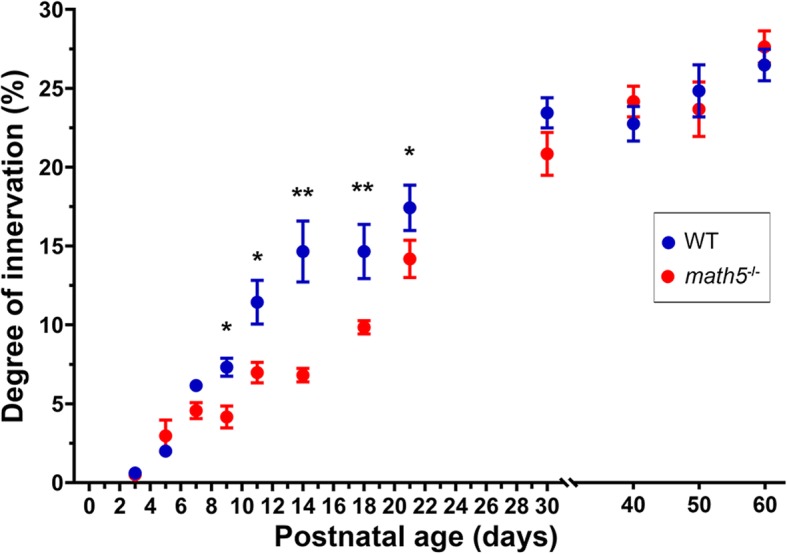
Summary plot depicting the degree of cholinergic innervation in dLGN, as a function of postnatal age in WT (blue) and *math5*^−/−^ (red) mice. Each data point represents the mean (±SEM) derived from a total of 4–6 hemispheres, using 3–5 successive sections through the middle of dLGN. Asterisks indicate statistically significant differences in cholinergic innervation between WT and *math5*^−/−^ (* *p* < 0.05, ** *p* < 0.01)

To compare the rate of cholinergic innervation in the WT and *math5*^−/−^ mice, we quantified the degree of cholinergic innervation as the percentage of fluorescent pixels in relation to the total number of pixels within dLGN, as a function of postnatal age. Figure 5 presents mean values for WT and *math5*^−/−^ mice between P3-P60. While both groups showed a progressive increase with age between P5–30, the rate of innervation was slower for *math5*^−/−^ between P9 and P21. At these ages, the degree of innervation was significantly lower in *math5*^−/−^ compared to age-matched WTs (two-way ANOVA, *F* = 2.49, Bonferroni *post-hoc* test, P9, *p* < 0.05; P11, *p* < 0.01; P14, *p* < 0.01; P18, *p* < 0.01; P21, *p* < 0.05). At P30, the degree of innervation was similar in both groups and remained stable into adulthood (P30–60).

**Fig. 6:**
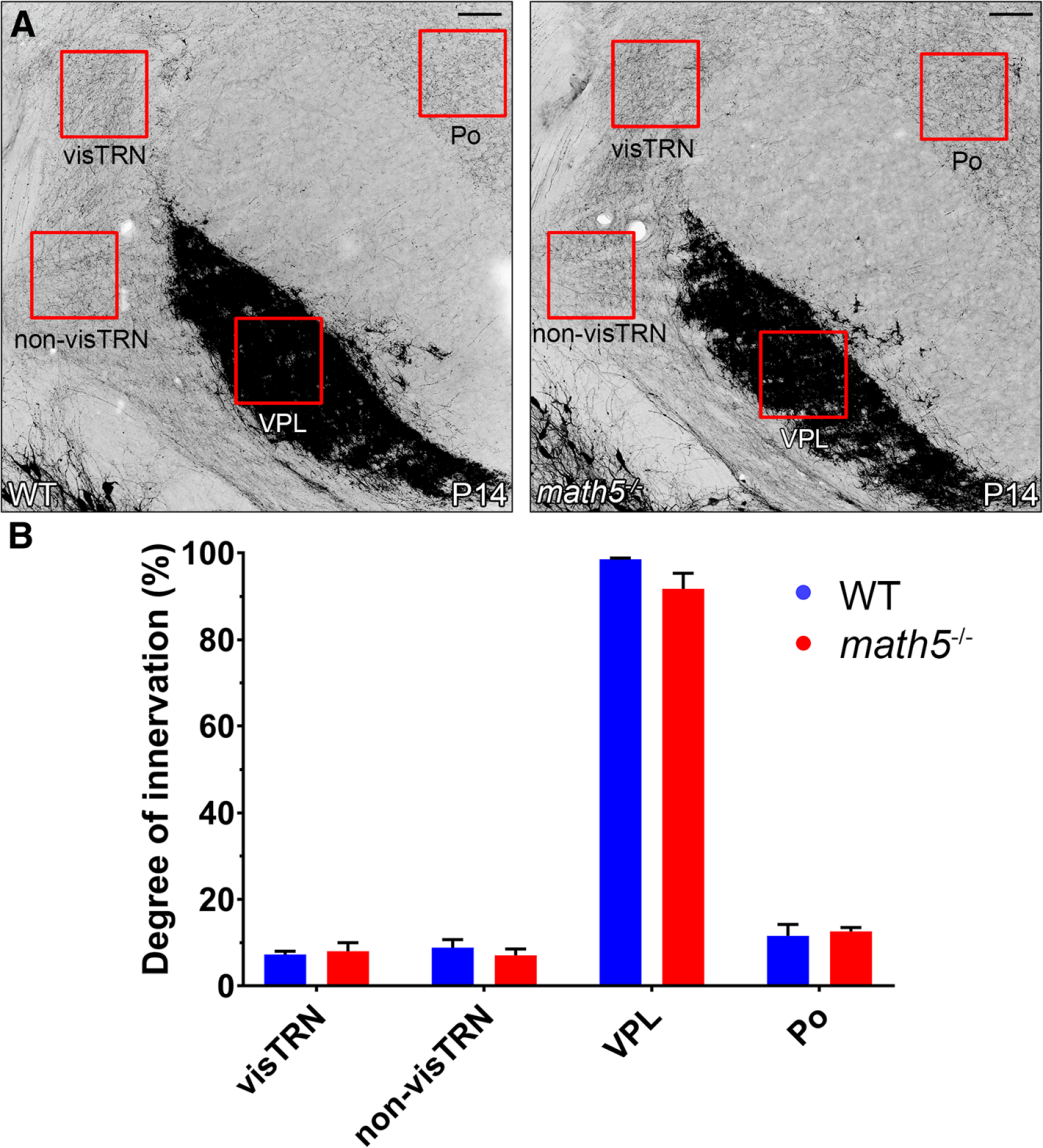
Cholinergic innervation of the visual and non-visual sectors of the thalamic reticular nucleus (TRN), the ventral posterolateral nucleus (VPL), and posterior nucleus (Po) in WT and math5^−/−^ mice at P14. **a** Examples are coronal sections through the dorsolateral aspect of thalamus. Boxes depict regions of interest where the extent of cholinergic innervation was measured. Scale bars = 100 μm. **b** Summary plot depicting the degree of cholinergic innervation in TRN, VPL, and Po for WT (blue) and *math5*^−/−^ (red) mice. Each bar represents the mean (±SEM) derived from a total of 6 hemispheres, using 3–5 successive sections

To rule out the possibility that *math5*^−/−^ leads to a more generalized disruption in brainstem cholinergic innervation of thalamus, we examined the degree of cholinergic innervation in nuclei that do not receive direct retinal input. These include the visual and non-visual sectors of the thalamic reticular nucleus (visTRN and non-visTRN), the ventral posterolateral nucleus (VPL), and posterior nucleus (Po) of the thalamus. Figure 6 presents examples of coronal sections through these nuclei at P14 (the time of peak cholinergic disruption in dLGN, Fig. 5) for WT (Fig. 6a, left) and *math5*^−/−^ mice. (Fig. 6a, right). The squares represent a 200 × 200 μm region of interest where measurements of cholinergic innervation were obtained. Figure 6B summarizes these comparisons and shows that for each region, the degree of innervation between WT and *math5*^−/−^ was comparable (Student’s T-test, visTRN t(6) = 0.348, *p* = 0.740, non-visTRN t(6) = 0.372, *p* = 0.762, VPL t(6) = 1.912, *p* = 0.104, Po t(6) = 0.372, *p* = 0.723).

**Fig. 7:**
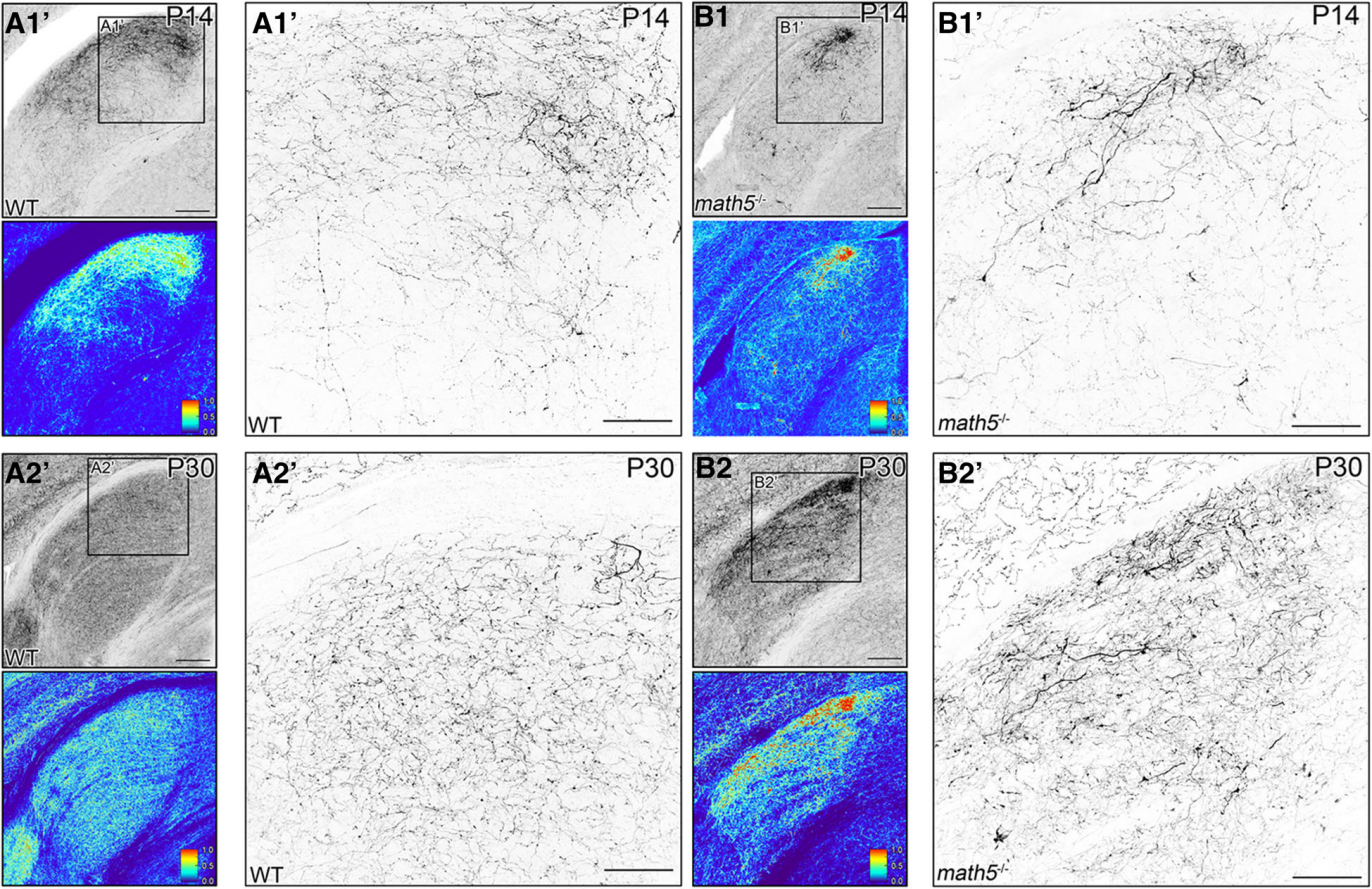
Terminal arbor organization of cholinergic fibers in dLGN in WT and *math5*^−/−^ mice. Examples are coronal sections through dLGN of WT (A1–2) and *math5*^−/−^ (B1–2) mice at P14 and P30. Top left panels depict a low power image of dLGN. Bottom left panels are corresponding heat maps of fluorescence intensity, where red and blue colors correspond to relative signal intensity values, highest values in red, and lowest values in blue. Right panels show a high power view of the corresponding insets. *A1–2.* In P14 WT (A1), cholinergic fibers form a homogenous pattern of fluorescence in the dorsolateral sector of dLGN, which at P30 (A2) extends throughout the entire nucleus as innervation reaching adult levels. *B1–2.* In P14 (B1) and P30 (B2) *math5*^−/−^ mice, the absence of retinal input leads to disruption of cholinergic terminal arbor organization forming ectopic patches of intense labeling consisting of thick, tangled axonal processes. Scale bars = 100 μm (left panels), 50 μm (right panels)

The architecture of cholinergic arbors in dLGN of *math5*^−/−^ mice also differed from WT. Examples of these differences are illustrated in Fig. 7 which depicts low and high power views of cholinergic projections in WT (Fig. 7A1 & A2) and *math5*^−/−^ (Fig. 7B1 & B2) mice at P14 and P30. In WT, low power images along with a corresponding “heat map” that plots the intensity of tdT fluorescence, revealed a diffuse and a relatively homogeneous intensity profile for areas of cholinergic innervation. High power views of these regions showed an orderly arrangement of fine-caliber axonal processes. By contrast, *math5*^−/−^ mice exhibited ectopic patches of intense fluorescence consisting of a dense tangled plexus of processes.

**Fig. 8:**
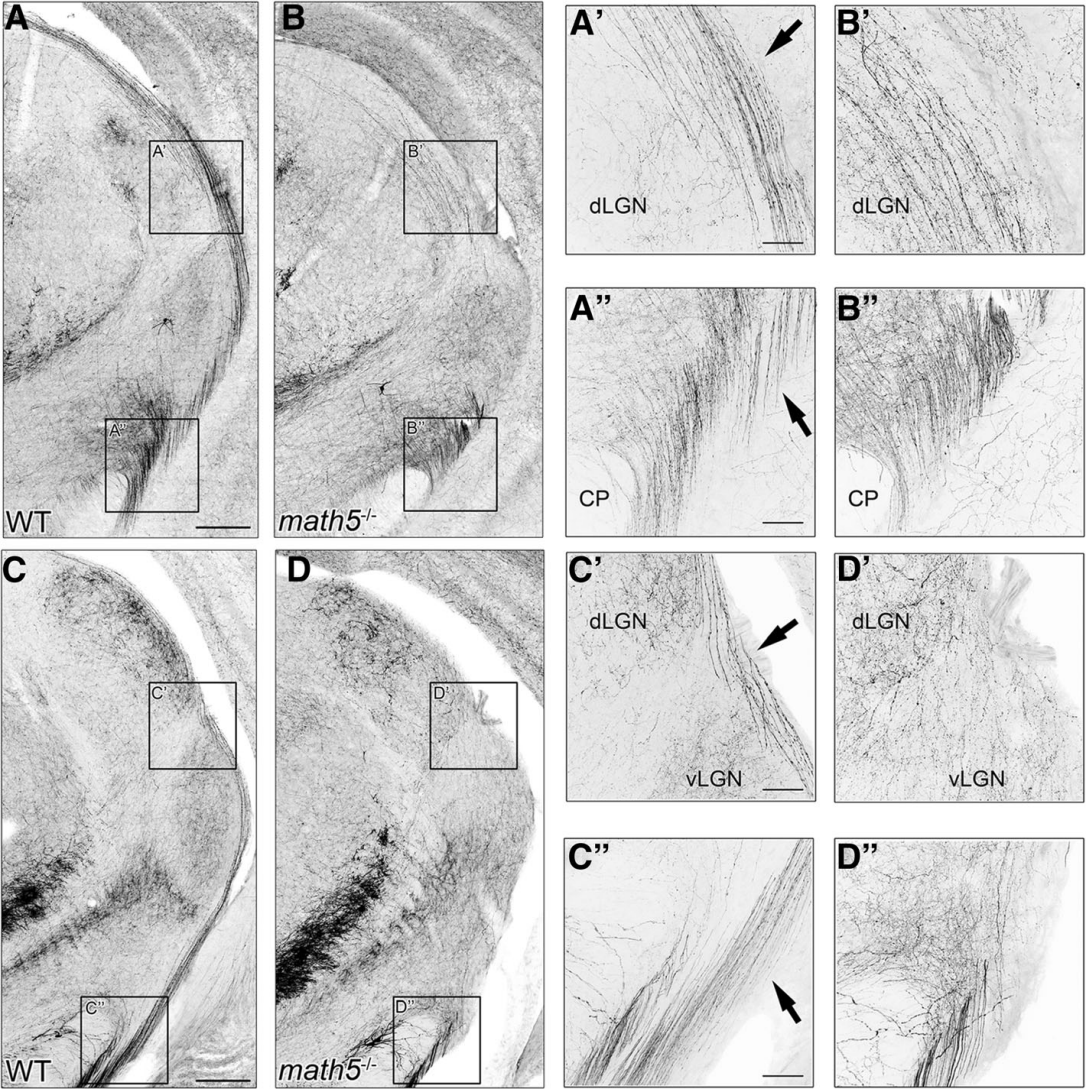
Routing of optic tract-traversing cholinergic axons WT and *math5*^−/−^. Examples of coronal sections through the thalamus are shown at the level of caudal dLGN (top panels) and middle dLGN (bottom panels) for WT (A & C) and *math5*^−/−^ (B & D). High power views at the level of the cerebral peduncle and the border of dLGN/vLGN are shown in the right hand panels. In WT (A & C), cholinergic axons travel with the optic tract near the cerebral peduncle (A” & C″), and continue dorsally along the outer border of the vLGN and dLGN (A’ & C′). In *math5*^−/−^ (B & D), cholinergic axons emerge near the cerebral peduncle (B″ & D”), but are displaced medially in the caudal dLGN (B′) and middle dLGN (D’). Scale bars = 200 μm (left panels), 50 μm (right panels)

Another salient phenotype in all *math5*^−/−^ mice was an alteration in the trajectory of cholinergic axons, especially those which normally traverse through the dorsal thalamus along the optic tract. Figure 8 shows coronal sections of the thalamus in WT and *math5*^−/−^ mice (P14) at a caudal (Fig. 8a and b) and rostral (Fig. 8c and d) level of dLGN. In WTs (Fig. 8a and c), at both levels a large number of cholinergic axons were seen traveling along the outer border of the thalamus. High power views show these projections arise with the optic tract near the cerebral peduncle (Fig. 8A” & C″), and run dorsally along the lateral border of vLGN and dLGN (Fig. 8A’ & C′). However, in *math5*^−/−^ mice, cholinergic axons were displaced medially at caudal dLGN (Fig. 8B & B’), and were not readily apparent at a more rostral level (Fig. 8D & D’). Moreover, while cholinergic axons were present near the cerebral peduncle (Fig. 8B”), they failed to fasciculate into separate bundles (Fig. 8D”), that in WT course in the supraoptic decussation and in the optic tract.

**Fig. 9:**
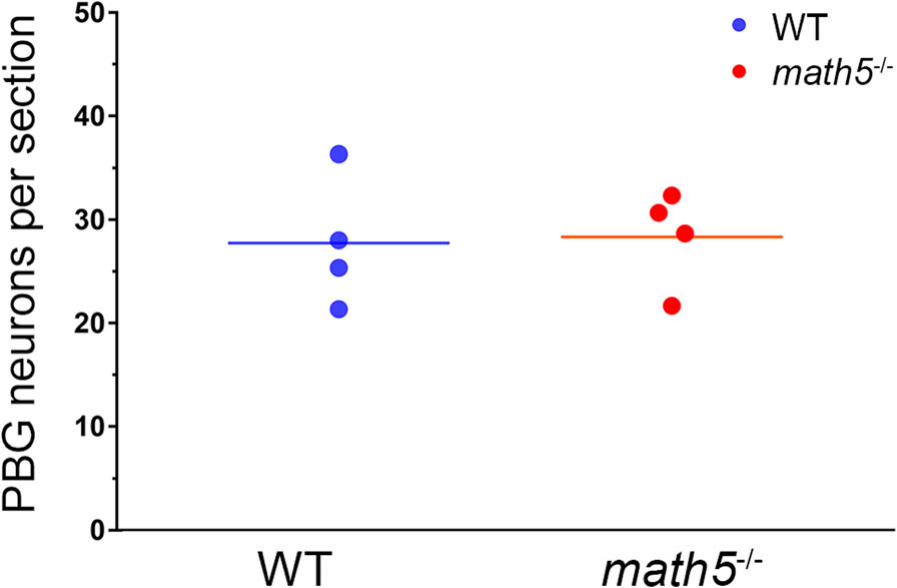
Plot showing the number of tdT-labeled neurons in PBG of WT and *math5*^−/−^ ChAT-Cre x Ai9 mice. Each point represents a single hemisphere. The values are dervied from the average of three sections taken through the middle of PBG. The horizontal lines depict mean group values

Previous studies in rodents indicate that cholinergic axons within the optic tract arise from PBG [20, 40]. To determine whether PBG is the source of the misrouted cholinergic axons observed in *math5*^−/−^, we selectively labeled PBG neurons in adult ChAT-Cre (WT, *n* = 3) and ChAT-Cre *x math5*^−/−^ (*math5*^−/−^, *n* = 4) mice using injections of Cre-dependent anterograde adeno-associated viral tracer FLEX-AAV-ChIEF-tdTomato. Before conducting viral tracing experiments, we used ChAT-Cre x Ai9 reporter lines to determine whether the number of PBG neurons is affected by the deletion of *math5*. Figure 9 depicts a plot showing the number of PBG neurons in WT and *math5*^−/−^ ChAT-Cre x Ai9 mice. Estimates reveal no significant differences between the two groups (Student’s t-test, t(6) = 0.148, *p* = 0.887). On average, we found about 27 tdT labeled neurons in a sample of sections through the middle of PBG for both groups.

**Fig. 10:**
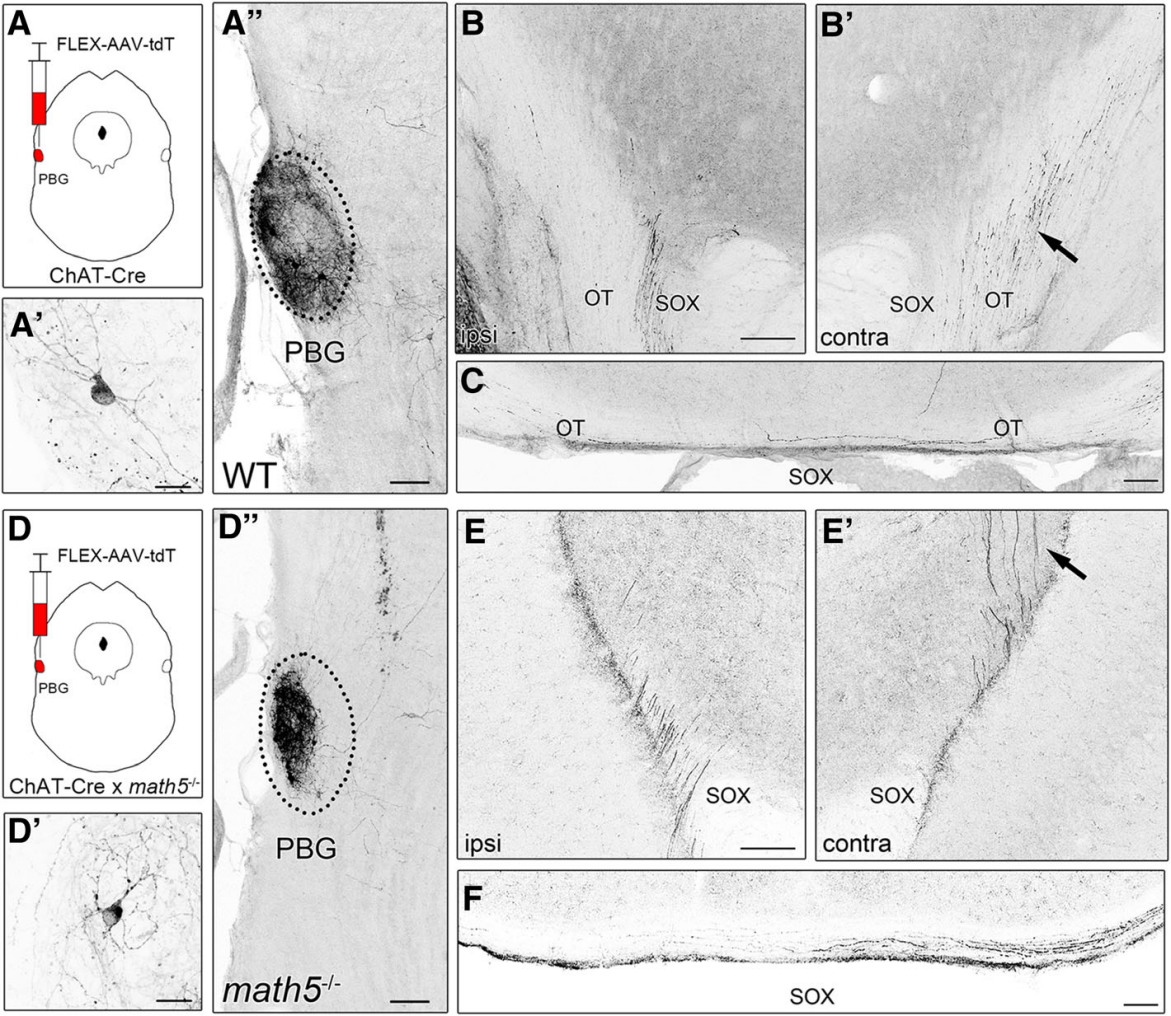
Trajectory of cholinergic PBG axons in the WT and *math5*^−/−^ mice. Examples show a unilateral viral tracer (FLEX-AAV-ChIEF-tdT) injection into PBG of an adult WT (A-C) and *math5*^−/−^ (D-F) ChAT-Cre mice, and the corresponding axonal labeling in the supraoptic decussation (SOX) at the level of cerebral peduncle (CP) and ventral midline. **a** Injection of viral tracer in WT ChAT-Cre mouse resulted in robust tdT labeling of neurons (A’), restricted to the left PBG (A”). **b** View of the cerebral peduncles, showing PBG axons traveling in the ipsilateral supraoptic decussation (B), and running dorsally in the contralateral optic tract (B′, arrow). **c** View of the ventral midline showing PBG axons crossing in the supraoptic decussation and running in the optic tract. **d** Injection of the viral tracer in *math5*^−/−^ mouse resulted in robust tdT labeling of neurons of the left PBG. **e** In *math5*^−/−^ mice, PBG axons travel in the supraoptic decussation ipsilaterally (E), and run dorsally within the neuropil of the contralateral thalamus (E’, arrow). **f** View of the ventral midline showing PBG crossing in the supraoptic decussation and running laterally along the ventral aspect of the brain. Scale bars = 100 μm, 20 μm (A’ & D’)

**Fig. 11:**
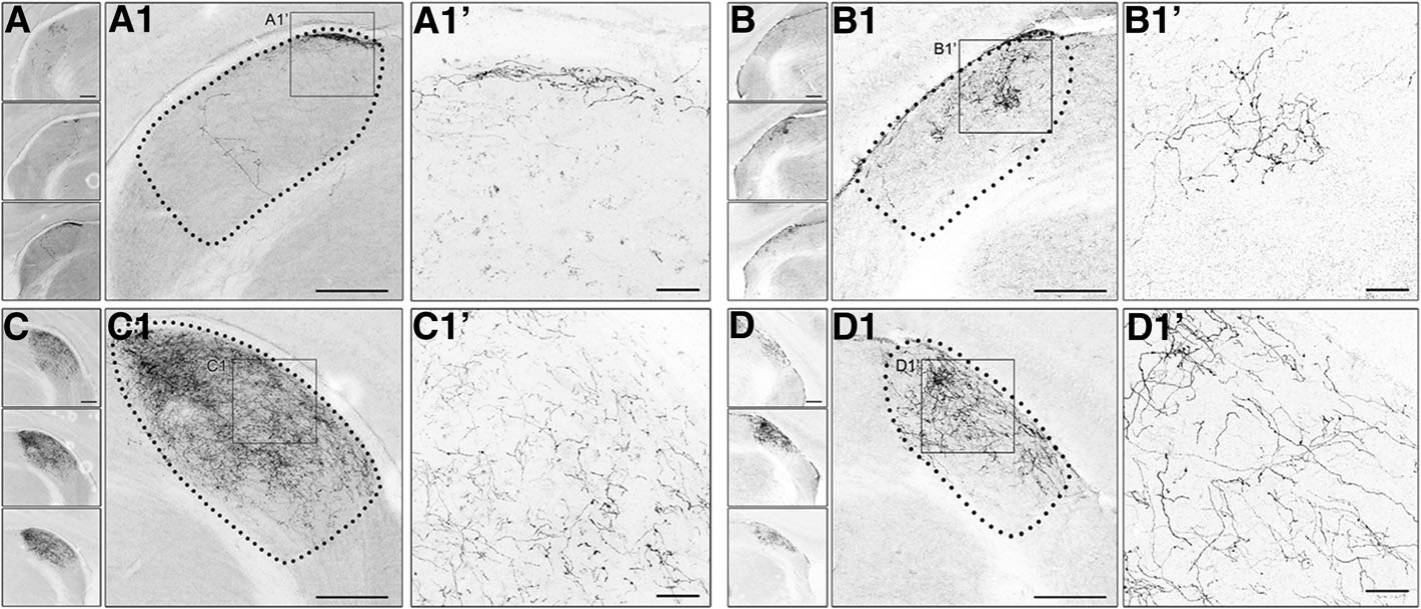
The pattern of PBG terminal arbors in dLGN in the presence and absence of retinal input (*math5*^−/−^). Examples of coronal sections depicting tdT-labeled terminal arbors in dLGN after an injection of FLEX-AAV-ChIEF-tdT virus into the left PBG in a WT (A & C) and *math5*^−/−^ (B & D) ChAT-Cre mouse. Left panels depict low power images, showing the distribution of PBG arbors in caudal (top panel), middle, and rostral (bottom panel) dLGN. Middle panels show a higher power view depicting PBG arbor distribution within an example section of dLGN. Dotted lines delineate the boundaries of dLGN. Right panels show high power views of PBG arbors. **a** In ipsilateral dLGN of WT, arbors are restricted to a small region in the dorsomedial region of the middle and rostral dLGN, just beneath the optic tract (A1 & A1’). **b** In the ipsilateral dLGN of *math5*^−/−^, PBG arbors are were displaced into the medial regions of the nucleus (B1 & B1’). **c** In the contralateral dLGN of WT, arbors are distributed throughout the caudo-rostral extent of dLGN (C1, C1’). **d** In the contralateral dLGN of *math5*^−/−^, arbors are distributed throughout the caudo-rostral extent. Scale bars = 200 μm (left panels) and middle 30 μm (right panels)

Figure 10 shows the pattern of axonal projections following the unilateral labeling of PBG in a WT (Fig. 10a) and *math5*^−/−^ (Fig. 10d) mouse. Examples of the injection site in PBG for WT and math5^−/−^ are shown in Fig. 10A’ and 10D’, along with high power views of tdT labeled PBG neurons in Fig. 10A” and D”. Dense labeling was seen throughout much of the nucleus, and estimates of somatic labeling within the middle three sections of PBG ranged between 10 and 27 neurons per case. In WT, PBG axons coursed within the ipsilateral supraoptic decussation near the cerebral peduncle (Fig. 10b), crossed to the contralateral hemisphere at ventral midline (Fig. 10c), and ascended along the lateral aspect of thalamus with the optic tract (Fig. 10B’). In *math5*^−/−^, PBG axons followed a similar trajectory (Fig. 10E & F) until reaching contralateral ventral thalamus, where they veered in a medial direction traversing dorsally through the medial geniculate nucleus and vLGN (Fig. 10E’). Figure 11 illustrates the pattern of cholinergic arbors within dLGN of WT and *math5*^−/−^ mice after a unilateral injection into PBG. In WT, the projection to the ipsilateral dLGN was restricted to a small strip of arbors in the dorsomedial pole (Fig. 11A, A1, & A1’). By contrast these projections were more variable in *math5*^−/−^, either missing altogether (*n* = 2) or showing substantial spread beyond the dorsomedial pole of dLGN. The projections in contralateral dLGN appeared to be similar in both WT and *math5*^−/−^, with arbors distributed more evenly throughout the nucleus (Fig. 11C, & Fig. 11D).

**Fig. 12:**
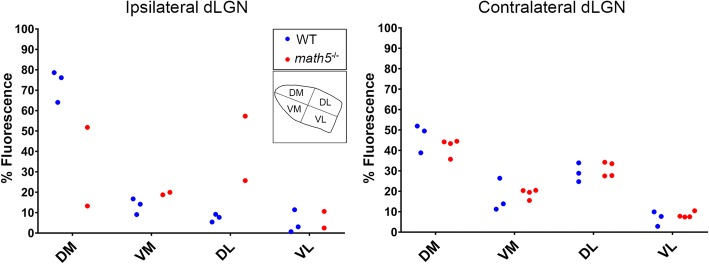
Plot comparing the distribution of tdT-labeled PBG arbors in dLGN of WT and *math5*^*−*/−^ mice after a unilateral injection of FLEX-AAV-ChIEF-tdT into PBG. Inset depicts the division of dLGN into 4 quadrants; dorsomedial (DM), ventromedial (VM), dorsolateral (DL), and ventrolateral (VL). Values in each quadrant reflect a percentage of the total fluorescence detected throughout the entire nucleus. Each point depicts the average value taken from 4 to 5 sections of a single hemisphere. Two *math5*^*−*/−^ mice lacked an ipsilateral projection, and are not shown

To compare the pattern of PBG projections in WT (*n* = 3) and *math5*^−/−^ (*n* = 4) ChAT-Cre mice, we quantified the distribution of tdT-labeled arbors within dLGN. Figure 12 shows a plot of the spatial distribution of tdT-labeled PBG fibers in the ipsilateral (left) and contralateral (right) dLGN. Values represent the percentage of fluorescence in each of four quadrants (Fig. 12 inset: dorsomedial, DM; ventromedial, VM; dorsolateral, DL; ventrolateral, VL). In the ipsilateral dLGN of WT, we found a significant difference in fiber distribution between quadrants (Kruskal-Wallis, *H* = 8.23, *p* = 0.014), with the DM quadrant exhibiting the highest proportion of cholinergic labeling (mean = 72.9%). In the ipsilateral dLGN of *math5*^−/−^, the distribution of labeling was variable across the quadrants (*n* = 2), or absent altogether (*n* = 2). It is important to note despite being absent in two cases, the corresponding contralateral labeling pattern was consistently present and no different from other mice. Since all injections were of the same volume and restricted to PBG, the absence of ipsilateral projections may simply reflect inherent variability brought about by absence of retinal input. In the contralateral dLGN of WT mice, there was a significant difference between quadrants (K-W, *H* = 9.97, *p* = 0.0003), with values in DM higher than VL quadrant (Dunn’s *post-hoc* test, DM vs. VL, *p* = 0.013). A similar pattern was noted in the contralateral dLGN of *math5*^−/−^ (K-S, *H* = 14.12, *p* < 0.0001, Dunn’s *post-hoc* test, DM vs. VL; *p* = 0.002). Finally, arbor distribution in the contralateral dLGN of WT and *math5*^−/−^ were not significantly different from each other (two-way ANOVA, *F* = 0.87, *p* = 0.47).

## Discussion

We used a ChAT-Cre mouse, crossed to a tdTomato reporter line (Ai9), to visualize and track the development of brainstem cholinergic projections to dLGN. Our results reveal that cholinergic brainstem innervation of mouse dLGN begins at the end of the first postnatal week, and increases slowly with age to reach an adult-like density by the end of the first postnatal month. Initially, cholinergic fibers appear in the dorsolateral region of dLGN just beneath the optic tract, then gradually progress in a ventromedial direction to form a homogeneous plexus of fibers throughout dLGN. This is consistent with earlier studies in cat and mouse, showing a slow but steady increase in ChAT immunoreactivity with postnatal age [17, 18]. Thus, cholinergic innervation of dLGN occurs well after the establishment of the retinogeniculate pathway [1]. Moreover, our results support the view that innervation of dLGN occurs in a sequential manner, with retinal projections arriving before nonretinal ones [9].

Although corticothalamic and cholinergic brainstem projections begin to arrive in dLGN at roughly the same time (P5), their rate of innervation is vastly different. For example, cortical innervation of dLGN is nearly complete between P9–12 [8, 9], yet brainstem cholinergic innervation is still sparse and restricted largely to the dorsolateral region, only reaching adult-like density of innervation by the end of the first postnatal month. Such timing indicates that the reciprocal connections between the dLGN and visual cortex are established prior to cholinergic brainstem input. The late postnatal onset of cholinergic brainstem innervation is consistent with the maturation of other reticular ascending arousal systems [41–44]. Overall this suggests that network-wide, state-dependent modulation of sensory information becomes operational well after sensory connections are established.

Our results in *math5*^−/−^ mice also support earlier studies underscoring the importance of retinal input in regulating the timing of nonretinal innervation of dLGN [9]. The absence of retinal input slowed the rate of cholinergic innervation to dLGN, though the overall degree of innervation measured in adults was preserved. It is important to note that retinal input regulates timing of nonretinal input in a bidirectional manner. Whereas the current study showed a slower rate of cholinergic innervation of dLGN in *math5*^−/−^ mice, previous studies in the same mutant demonstrate an acceleration of corticogeniculate innervation, suggesting that retinal input regulates nonretinal development through distinct molecular mechanisms. The accelerated arrival of corticogeniculate axons in *math5*^−/−^ mice results from the disruption of aggrecan, a repellant extracellular matrix molecule that prevents the premature entry of cortical axons into dLGN [11]. While the molecular mechanism underlying retinal regulation of cholinergic innervation in dLGN is unresolved, the disruption in the rate of cholinergic innervation in *math5*^−/−^ mice may reflect a reduction of trophic support during development. In other regions that receive cholinergic innervation, neurotrophins such as brain-derived neurotropic factor (BDNF) and neurotrophin-3 (NT-3), have been shown to promote the growth of cholinergic neurites [45–47]. In WT mice, BDNF and NT-3 are anterogradely transported from the retina to dLGN [48, 49]. Therefore, it is possible that a reduction in the levels of these factors may underlie the dystrophic growth of cholinergic fibers observed in *math5*^−/−^ mutants.

In addition to regulating the rate of cholinergic innervation of dLGN, we demonstrated that retinal input plays a role in establishing the thalamic trajectory of a subset of brainstem cholinergic axons. These axons, which arise from cholinergic neurons of PBG, normally run within the optic tract along the outer border of the thalamus en route to dLGN. However, in *math5*^−/−^ mice PBG axons are displaced, traveling in a diffuse manner through thalamus. This indicates that during development, PBG axons use the retinal axons of the optic tract as a scaffold to navigate through the thalamus. Such axon-axon interactions are typically mediated by cell-adhesion molecules, which promote growth along the length of an existing axon [50]. Surprisingly, our tracing studies in *math5*^−/−^ mice revealed that such interactions are not necessary for PBG axons to elongate and reach their appropriate target. We found that PBG axons continued to grow through thalamus and reach dLGN even in the absence of an optic tract. Furthermore, nuclei-specific targeting was preserved in *math5*^−/−^ mice. PBG axons continued to innervate dLGN (and superior colliculus, unpublished observations) and were not found in neighboring structures within the lateral geniculate complex. In addition, the laterality of the projection did not appear to be affected, as the bulk of PBG arbors targeted the contralateral dLGN in both WT and *math5*^−/−^ mice. Therefore, it is likely that PBG axons rely on cues expressed within the thalamus to target, innervate, and arborize in dLGN. Future experiments are needed to fully elucidate the specific cues that govern PBG axon guidance.

Though retinal input does not appear to be necessary for nuclei-specific targeting of PBG input, it plays an important role in establishing the appropriate pattern and morphology of arbors within dLGN. In WTs, PBG arbors were consistently found within the dorsomedial pole of the ipsilateral dLGN, adjacent to the optic tract. However in *math5*−/− mice, this pattern was perturbed, with arbors extending beyond their usual target or perhaps failing to reach the nucleus altogether. Interestingly, in both WT and *math5*^−/−^ mice, PBG projections to the contralateral dLGN were similar in distribution.

In several mammals, inputs from PBG, superior colliculus, and certain types of retinal ganglion cells converge to terminate in discrete regions of dLGN (e.g., C-laminae of carnivores, koniocellular layers of primates), suggesting the existence of a conserved visual channel involved in the coordination of visuo-motor processing [20, 21, 51–53]. The rodent dLGN contains a homologous region, known as the dorsolateral shell [54], which receives input from direction selective retinal ganglion cells (DSGCs), as well as the superficial layers of the superior colliculus [51, 55]. In WT mice, we found an ipsilateral PBG projection to dLGN that targets the dorsolateral shell but in a circumscribed area that appears to represent the upper nasal visual fields [56, 57]. While the presence of retinal input appears to be necessary for the proper targeting of PBG arbors to the ipsilateral dLGN it has little impact if any on the more widespread and diffusely organized contralateral projections. Thus, it is not yet clear how this is accomplished, or whether it involves interactions with other inputs that target the shell, such as those from DSGCs and/or the superior colliculus.

## Conclusions

The mouse dLGN has emerged as a useful model system for exploring the development of visual thalamic circuitry. However, relatively little is known about how and when nonretinal connections are formed, or whether their timing of innervation is regulated by retinal input. In this study, we examined the developmental time course of a major ascending nonretinal input to dLGN, cholinergic brainstem nuclei, by using a transgenic mouse line to visualize cholinergic input (ChAT-Cre), and a knockout line devoid of retinal input (*math5*^−/−^). We found that the cholinergic innervation has a protracted time course, innervating dLGN over the course of first postnatal month, with the bulk of input arriving well after retinal connections are formed. In *math5*^−/−^, we found that the rate of cholinergic innervation is slowed compared to WTs. Furthermore, the routing and pattern of arborization of optic tract-traversing PBG cholinergic axons was altered in the ipsilateral dLGN of *math5*^−/−^ mice. Our anatomical tracing data demonstrated that while retinal input is not necessary for PBG innervation of dLGN, it plays an important role in the organization of arbors within the nucleus.

## Abbreviations

AAV: Adeno-associated virus
AF: Alexa Fluor
ANOVA: Analysis of variance
BDNF: Brain-derived neurotrophic factor
ChAT: Choline acetyltransferase
CP: Cerebral peduncle
CTB: Cholera toxin subunit B
DL: Dorsolateral
dLGN: Dorsal lateral geniculate nucleus
DM: Dorsomedial
DSGC: Direction-selective ganglion cell
LDTg: Laterodorsal tegmentum
LP: Lateral posterior nucleus
NGS: Normal goat serum
non-visTRN: non-visual sector of thalamic reticular nucleus
NT-3: Neurotrophin-3
OT: Optic tract
OX: Optic chiasm
P: Postnatal
PBG: Parabigeminal nucleus
PBS: Phosphate-buffered saline
PCR: Polymerase chain reaction
PFA: Paraformaldehyde
Po: Posterior nucleus
PPTg: Pedunculopontine tegmentum
PT: Pretectum
RGC: Retinal ganglion cell
SC: Superior colliculus
sox: Supraoptic decussation
tdT: TdTomato
TRN: Thalamic reticular nucleus
visTRN: Visual sector of thalamic reticular nucleus
VL: Ventrolateral
vLGN: Ventral lateral geniculate nucleus
VM: Ventromedial
VPL: Ventral posterolateral nucleus
WT: Wild type
